# Plasma attenuates endothelial injury compared to crystalloids in a ventilated rat pneumosepsis model

**DOI:** 10.1371/journal.pone.0319272

**Published:** 2025-02-25

**Authors:** Daan P. van den Brink, Derek J.B. Kleinveld, Chantal A. Polet, Hendrik Veltman, Joris J.T.H. Roelofs, Nina C. Weber, Nicole P. Juffermans

**Affiliations:** 1 Department of Intensive Care Medicine, Amsterdam UMC, University of Amsterdam, Amsterdam, Netherlands; 2 Laboratory of Experimental Intensive Care and Anesthesiology, Amsterdam UMC, University of Amsterdam, Amsterdam, Netherlands; 3 Department of Anesthesiology, Erasmus MC, Erasmus University of Rotterdam, Rotterdam, Netherlands; 4 Department of Pathology, Amsterdam UMC, University of Amsterdam, Amsterdam, Netherlands; 5 Amsterdam UMC, Cardiovascular Sciences, Amsterdam, The Netherlands; 6 Department of Intensive Care Medicine, Erasmus MC, Erasmus University of Rotterdam, Rotterdam, Netherlands; University of South Carolina, UNITED STATES OF AMERICA

## Abstract

**Background:**

The dysregulated immune response during sepsis involves endothelial injury, which may be augmented by infusion of clear fluids such as crystalloids. Plasma has been suggested as an alternative resuscitation fluid but it is unclear whether previously observed benefits were due to the type of fluid, or due to less volume required to restore tissue perfusion. We hypothesized that resuscitation with plasma reduces endothelial injury, inflammation, and organ injury compared to similar and higher volumes of crystalloids in a rat pneumosepsis model.

**Methods:**

Rats were intratracheally inoculated with Streptococcus Pneumoniae to induce pneumosepsis. Twenty-four hours after inoculation, animals were randomized to 4 groups: healthy controls (non-resuscitated, n = 6), 10 ml/kg/hr (standard-volume, n = 11) crystalloid resuscitation, 3.33 ml/kg/hr (low-volume, n = 11) crystalloid resuscitation or 3.33 ml/kg/hr plasma resuscitation (n = 11). Plasma markers of inflammation and endothelial injury were measured. Organs were harvested for histology and wet-to-dry weight ratio determination.

**Results:**

Inoculated animals developed pneumosepsis, with lower mean arterial pressures (p < 0.001) and higher lactate levels (p < 0.001) compared to healthy controls. Animals resuscitated with plasma showed a trend towards lower syndecan-1 levels compared to the standard-volume crystalloid group (82 vs 99 ng/mL, p = 0.06) and had lower levels of VCAM-1 (424 vs 592 ng/mL, p < 0.01) compared to the standard volume crystalloid group, but not when compared to the low-volume crystalloid group. Other markers of endothelial injury or inflammation were not significantly different between groups. No significant differences were observed in histologic injury scores and wet-to-dry ratios.

**Conclusion:**

Plasma resuscitation modestly reduces endothelial injury compared to crystalloid resuscitation. This effect might be attributed to decreased resuscitation volumes rather than the type of fluid.

## Introduction

Sepsis is characterized by a dysregulated inflammatory host response, which involves endothelial injury [1,2]. The glycocalyx is a gel-like layer covering the luminal surface of the vessel wall [3]. During sepsis, glycocalyx constituents, including syndecan-1 and thrombomodulin, are released into the circulation [4]. Degradation of the glycocalyx contributes to endothelial permeability [5], microthrombi formation and augmentation of the inflammatory response [6–8], which collectively worsens patient outcome [9,10]. Moreover, neutrophil activation leads to release of their chromatin structures termed Neutrophil Extracellular Traps (NETs), containing myeloperoxidase (MPO) and citrullinated histone H3 (CH3). NET formation facilitates the elimination of invading bacteria [11]. However, dysregulated NET formation can contribute to immunothrombosis, thereby contributing to endothelial and organ injury [12,13]. Therefore, strategies aiming to prevent endothelial injury and uncontrolled NET formation are thought to have the potential to reduce organ injury and improve patient outcomes [14].

Initial treatment of sepsis includes fluid resuscitation, aiming to restore adequate tissue perfusion. The Surviving Sepsis Campaign guideline suggests resuscitating septic patients with at least 30 mL/kg within the first 3 hours, recommending crystalloids as the primary resuscitation fluid [15]. However, low-protein fluids, such as crystalloids, may augment glycocalyx degradation, as shown *in vitro* [16]. Plasma may be a superior alternative fluid by preserving the endothelial barrier and reducing inflammation due to a protein-rich content, or due to specific factors [16,17]. *In vitro*, protein-rich solutions were found to inhibit Lipopolysaccharide (LPS)-induced NET formation [18]. Also, plasma may reduce microthrombosis, as found in a traumatic shock model [19]. In animal models of shock, plasma was superior in maintaining glycocalyx thickness and endothelial barrier function when compared to crystalloids [20–25]. However, in the experimental sepsis models, two-threefold higher volumes of crystalloids were used as compared to plasma [23–25]. This is probably due to the high protein content of plasma, increasing colloid osmotic pressure more effectively, with interstitial fluid reabsorption into the intravascular space. Thereby, it remains unclear whether the observed benefits of plasma resuscitation in these models were due to the fluid type or to less fluid volume. Of note, in our previous mild sepsis model, when comparing equal resuscitation volumes of plasma compared to crystalloids, we found that plasma did not reduce endothelial and organ injury [26]. However, in this model, low resuscitation volumes were used and recipients were not in shock.

In this study, we used a severe pneumosepsis rat model supported by mechanical ventilation to investigate the effects of resuscitation using a standard-volume of crystalloids (30 ml/kg in the first 3 hours) compared to lower-volume (10 ml/kg in the first 3 hours) of crystalloids and an equal low-volume of plasma (10 ml/kg in the first 3 hours). We hypothesized that plasma reduces inflammation, endothelial injury, and organ damage compared to both the standard and low-volume crystalloid groups, with a more profound beneficial effect when compared to the standard-volume crystalloid group.

## Materials and methods

Approval for this study was obtained from the Animal Care and Use Committee of the Amsterdam University Medical Centers, location AMC, University of Amsterdam, the Netherlands (project license: AVD1180020174327, study title: intervention on endothelial function in ventilated septic rats, approval date: 13-12-2022). All procedures adhered to the European Parliament Directive (2010/63/EU) and national law of the Experiments on Animals Act (Wod, 2014). In total, 39 male Sprague-Dawley rats (Envigo, Indianapolis, IN, USA), 12-14 weeks of age, weighing approximately 400 grams, were included. Rats were group-housed in standard cages with a 12-hour light-dark cycle for at least 7 days prior to the experiment to acclimatize. During this period, food and water were provided *ad libitum*. The study was conducted and reported in accordance with the ARRIVE guidelines (S3 Appendix) [27].

### Preparation of plasma product

Fresh frozen plasma (FFP) donor products from syngeneic rats were prepared in accordance with blood banks standards as described previously [26]. Briefly, 12–14-week old syngeneic donor rats were anesthetized using 5% isoflurane/95% FiO_2_ mix. Whole blood was collected via cardiac puncture using a 19G syringe in tubes containing 10% citrate-phosphate-dextrose solution (C165, Sigma-Aldrich, Saint Louis, MO, USA). To retrieve platelet-free plasma, immediately after retrieval, whole blood was centrifuged at 2000G, 18°C, acceleration 9, deceleration 3 for 15 minutes (5804R, Eppendorf AG, Hamburg, Germany). Thereafter, plasma was separated and centrifuged at 2000G, 4°C, acceleration 9, deceleration 3 for 15 minutes after which the upper 2/3^rd^ of plasma was taken and snap-frozen in liquid nitrogen. All FFP products were processed within 1 hour and stored at -80°C for future use.

Before freezing, arterial blood gas (ABG) (RAPIDPoint 500, Siemens, Germany) and blood cell count (Cell Coulter AcT diff2 Hematology Analyzer, Beckmann, Germany) were done on all plasma products to ensure quality and that products were platelet-free. During study days, FFP products were thawed at 37°C using a water bath and filtered through a Puradisc FP 30 mm, 0.2 µm filter (Whatman; GE Healthcare Life Sciences, Eindhoven, the Netherlands) before transfusion.

### Preparation of inoculum

Inoculums were prepared as described previously [26]. In short, *Streptococcus pneumoniae* (serotype 3, ATCC 6303; Rockville, MD, USA) were thawed from a frozen stock and cultured on Colombian blood agar containing 5% sheep blood plates (Biomerieux Benelux B.V., Zaltbommel, the Netherlands). Afterwards, they were transferred to brain-heart infusion broth (Amsterdam UMC, Amsterdam, the Netherlands) for overnight incubation at 37°C. Using a spectrophotometer (Secoman S250), the optical density (OD) was measured. Assuming an OD_620_ of 0.350 contains 1 * 10^8^ colony forming units (CFU) per ml, the final inoculum was prepared by diluting the suspension with sterile 0.9% saline solution. For all inocula, serial 10-fold dilutions were cultured on plates overnight to quantify administered CFU.

### Pneumosepsis model

**Fig 1:**
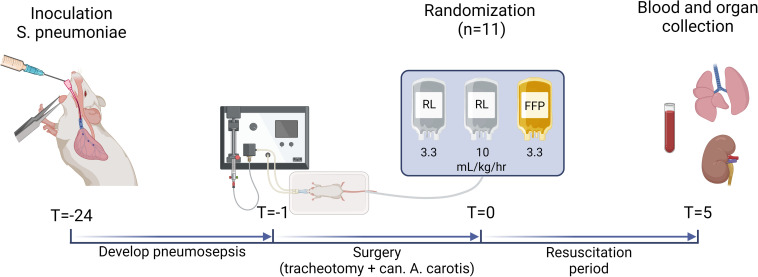
Schematic overview of the pneumosepsis model. Animals were intratracheally inoculated with sham inoculate or 3−5 x 10^8^ S. pneumoniae, after which they developed pneumosepsis. Twenty-three hours after inoculation (T = −1), animals were anesthetized and surgical procedures were performed. Twenty-four hours after inoculation, animals were randomized to resuscitation with standard-volume (10 ml/kg/hr) of Ringer’s lactate (RL), low-volume of RL (3.33 ml/kg/hr), or fresh frozen plasma (FFP) (3.33 ml/kg/hr). Products were made from syngeneic rat donors. Resuscitation was administered over a 5-hour period. After the resuscitation period, animals were exsanguinated and organs were collected. Of note, the used x-axis in this figure is not linear.

The experimental protocol is summarized in Fig 1. Twenty-four hours before resuscitation (T = −24), animals (n = 39) were anesthetized using 5% isoflurane/95% FiO_2_. Thereafter, 3-5 *  10^8^ CFU *S. pneumoniae* in 100 µL sterile 0.9% saline solution was intratracheally introduced (n = 33) to induce pneumosepsis. Healthy controls (n = 6) were inoculated with 100 µL sterile 0.9% saline solution. Afterwards, animals were transferred to their cages and allowed to wake up.

Twenty-three hours after inoculation (T = −1), an intraperitoneal bolus (1.5 mL/kg) containing R-ketamine (90 mg/kg), dexmedetomidine (0.125 mg/kg), and atropine (50 µg/kg) was administered. Thereafter, the tail vein was cannulated to administer maintenance anesthesia consisting of R-ketamine (50 mg/kg/hr) and dexmedetomidine (15 µg/kg/hr). After tracheostomy was conducted, rats were mechanically ventilated (VentElite, Harvard Apparatus, Holliston, MA, USA) with tidal volumes of 6 ml/kg, ventilator frequency of 55 bpm, inspiratory:expiratory ratio of 1:1.5, and FiO_2_ of 60%, which was based on pilot experiments. Lung recruitment maneuvers (inspiratory sigh of 20%) were performed every 30 minutes. The left carotid artery was cannulated to extract arterial blood samples and monitor mean arterial pressure (MAP). Rectal thermometers were placed to continuously monitor temperature. Body temperature was kept at 37°C using warmth mats and lamps.

Twenty-four hours after inoculation, after performing all surgical procedures (T = 0), septic animals were randomized with opaque envelopes using a 1:1 allocation to three different resuscitation groups: resuscitation using a standard-volume of 10 mL/kg/hr Ringer’s lactate (based on the recommendation of 30 mL/kg in the first 3 hours according to the Surviving Sepsis guidelines [15]) (standard-volume crystalloid group, n = 11), resuscitation using a low-volume of 3.33 mL/kg/hr Ringer’s lactate (low-volume crystalloid group, n = 11) and resuscitation using an equal low-volume of 3.33 mL/kg/hr plasma (low-volume plasma group, n = 11). Resuscitation was administered during a 5-hour period. Healthy controls received no resuscitation. Five hours after the start of resuscitation (T5), animals were terminated by exsanguination via cardiac puncture using a 19G needle. Lungs were removed for wet/dry ratios and histopathology. Exclusion criteria were negative blood and lung cultures and untimely death due to technical failures. Researchers were aware of group allocation from the moment of randomization.

### Animal welfare

Animal welfare was evaluated using a modified M-CASS rating system scoring visual characteristics including fur aspect, activity, behavior, face, diarrhea, and respiratory distress [23,28]. For each characteristic, a rating (1–4) was given right after inoculation, eight hours, and 24 hours after inoculation (Fig S4 in S1 Appendix). A score of 4 in any of the characteristics was considered a humane endpoint, resulting in the immediate termination and exclusion of an animal from the model. All personal evaluating animal welfare were trained in accordance with the requirements of article 9 or article 12 of the Experiments on Animals Act.

### Sample collection

During the experiment, mean arterial pressure (MAP), heart rate, body temperature, and oxygen saturation were measured continuously. Arterial blood samples were collected prior to resuscitation and at termination for blood gas analysis (RAPIDPoint 500, Siemens, Germany) and complete blood counts (Cell Coulter AcT diff2 Hematology Analyzer, Beckmann, Germany). Whole blood was processed as previously described [26].

### Assays

Biomarkers including Syndecan-1 (Elabscience, MD, USA), VCAM-1 (Elabscience, MD, USA), MMP-9 (Elabscience, MD, USA), ICAM-1 (R&D systems, MN, USA), IL-6 (R&D systems, MN, USA), IL-10 (R&D systems, MN, USA) and Citrullinated histone H3 (CH3) (Cayman Chemicals, MI, USA) were measured using commercially available ELISA kits according to manufacturer guidelines. Myeloperoxidase (MPO) was measured by adjusting a commercial cell death ELISA kit (Roche Diagnostics Nederland B.V., Almere, the Netherlands). For the capturing antibody, a myeloperoxidase polyclonal antibody (Pa5-16672, Invitrogen, MA, USA) was used. MPO data are shown as relative absorbance and were not quantified. Values of any ELISA that were lower than the reference value were set at the lowest detection range.

### Bacterial growth

The right inferior lobe of the lung was homogenized in 5:1 sterile 0.9% NaCl and plated on Colombian agar with 5% sheep blood plates in serial tenfold dilutions. Whole blood retrieved during exsanguination (T = 5) were plated on COS plates.

### Pulmonary histopathology

The right lung was promptly fixed in 10% formaldehyde after retrieval and then embedded in paraffin. 4-µm-thick sections were cut and stained with Haematoxylin and Eosin (H&E). A pathologist, who was blinded to treatment allocation, evaluated the tissue for the presence of edema, bronchitis, endothelialitis, interstitial inflammation and thrombi formation on a scale of 0–4 (0 = absent, 1 = mild, 2 = moderate, 3 = severe, 4 = very severe), as described previously [26]. The full scoring list can be found in the supplement (Table S1 in S1 Appendix).

### Sample size and statistical analysis

The primary outcome was syndecan-1 as a marker of glycocalyx degradation and endothelial injury. Based on a previous sepsis model, resuscitation with plasma compared to normal saline reduced syndecan-1 levels (21.8 vs 31.0 ng/mL) [23]. These data extrapolated to a sample size with 4 groups is used to correct for multiple testing with a power of 80%. A minimum of 10 rats per group were needed to find a statistically significant difference. A 5% mortality was expected. Thus, 11 rats per group were included.

All data were analyzed using SPSS Statistics V.28 IBM. Charts and graphs were designed in GraphPad Prism 9. All data were regarded as non-parametric and are shown as medians with interquartile ranges (IQR). Data were tested for differences using the Kruskal-Wallis test with post-hoc pairwise comparisons. A *p* value < 0.05 was considered statistically significant. All data collected until the moment of death was used in analyses.

## Results

### Pneumosepsis model and pre-resuscitation parameters.

**Fig 2:**
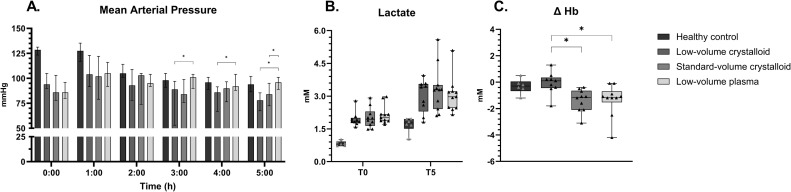
Mean arterial pressure, lactate and hemoglobin levels. Data are presented as median with interquartile ranges and minimum and maximum values showing all individual data points, Hb =  hemoglobin, * p < 0.05.

**Fig 3:**
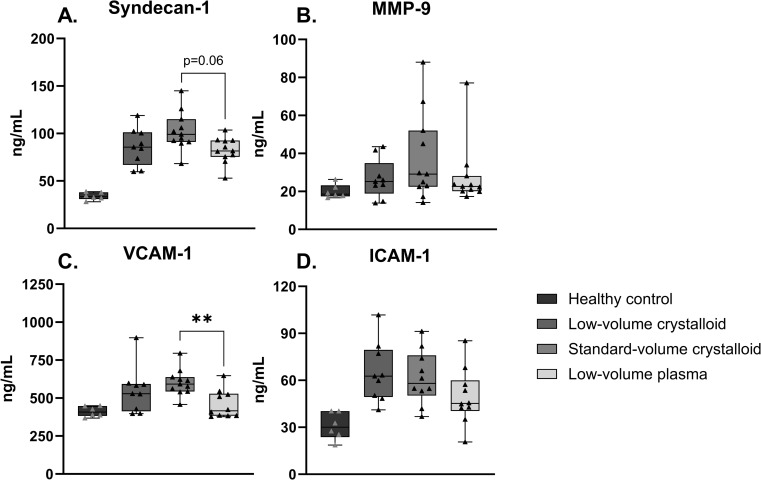
Markers of glycocalyx degradation and endothelial injury. Data are presented as median with interquartile ranges and minimum and maximum values showing all individual data points, **p < 0.01, ICAM-1 =  Intercellular Adhesion Molecule 1, MMP-9 =  Matrix metalloproteinase-9, VCAM-1 =  Vascular cell Adhesion Protein 1.

**Fig 4:**
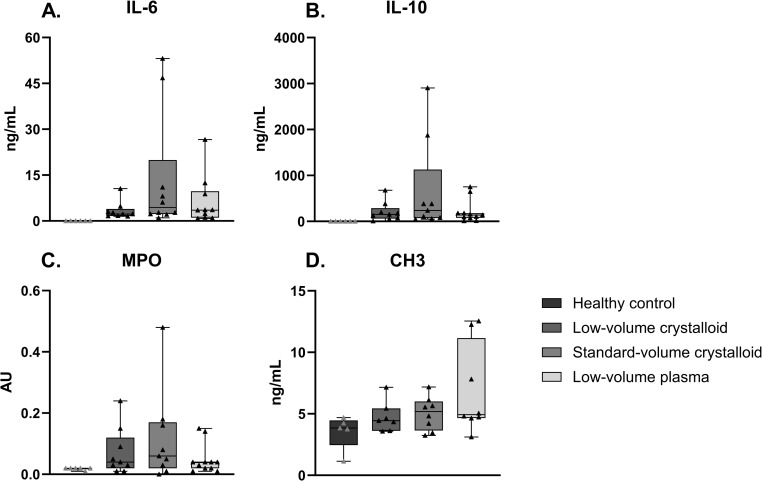
Inflammation and NET formation. Data are presented as median with interquartile ranges and minimum and maximum values showing all individual data points, CH3 =  Citrullinated Histone H3, IL =  interleukin, MPO =  myeloperoxidase.

**Fig 5:**
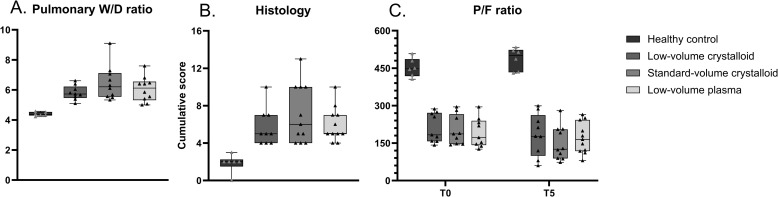
Markers of pulmonary injury. Data are presented as boxplots with median, interquartile ranges and minimum and maximum values showing all individual data points, W/D: Wet-to-dry ratio.

Inoculated male rats developed pneumosepsis, demonstrated by a decrease in mean arterial pressure (MAP) (median: 129 vs 93 mm Hg, p < 0.001, Fig 2), increased heart rates (HR) (228 vs 260 bpm, p < 0.001, Table S1 in S1 Appendix) and increased lactate levels (0.82 vs 1.91 mM, p < 0.001, Fig 2). Moreover, septic animals had weight loss, and significant lab alterations such as leucopenia and a drop in platelet counts (Table S1 in S1 Appendix). Severe lung injury was present, with reduced pulmonary function (P/F ratio: 443 vs 183, p < 0.001, Fig 5), lung edema (wet-to-dry ratio: 4.4 vs 5.9, p < 0.001) and increased pulmonary histologic injury scores (Fig 4). Also, sepsis resulted in renal edema (wet-to-dry ratio: 4.5 vs 5.2, p < 0.05), increased inflammation and endothelial injury with glycocalyx degradation (Figs 3 and 5). Between intervention groups, no baseline (T0) differences were seen (Fig 2, Table S1 in S1 Appendix).

### Resuscitation period, hemodynamics, and mortality

Throughout the experiment, two animals in the low-volume crystalloid group succumbed during the resuscitation period due to shock with decreasing blood pressures (Fig S1 in S1 Appendix). All other animals survived until the end of the study (N = 37). From 3 hours following resuscitation until sacrifice, rats receiving plasma maintained higher mean arterial pressures compared to the low-volume crystalloid group (p < 0.05) and had higher MAP than the standard-volume crystalloid group 5 hours post-resuscitation (p < 0.05) (Fig 2). Mean arterial pressure and heart rates were not different between the standard and low-volume crystalloid groups at any time point. Following the resuscitation period, lactate levels did not differ between all resuscitation groups (Fig 2). Hemoglobin levels were significantly decreased in animals receiving plasma or standard-volume crystalloid resuscitation compared to animals receiving low-volume crystalloid resuscitation, suggesting more effective intravascular volume expansion in these groups (Fig 2).

### Markers of glycocalyx degradation and endothelial injury

Rats receiving plasma compared to those receiving standard-volume crystalloid resuscitation, showed a trend towards lower syndecan-1 levels (median (IQR) 82 [75–93] vs 99 [91–115] ng/mL, p = 0.06) and had significantly lower levels of VCAM-1 (424 vs 592 ng/mL, p < 0.01) (Fig 3). ICAM-1 and MMP-9 were not significantly different between crystalloid and plasma groups (Fig 3).

### Inflammation and NET formation

Rats receiving plasma had no significant differences in levels of IL-6 (median [IQR] 3.6 [1.1–9.7] vs 6.1 [2.2–28.9] ng/mL, p = 0.54) and IL-10 (138.2 [77.1–179.1] vs 237 [70.3–1127.9] ng/mL, p = 0.46) compared to the standard-volume crystalloid group. Regarding NET formation, MPO (0.036 [0.015–0.042] vs 0.064 [0.023–0.174] AU, p = 0.29) and CH3 (4.9 [4.7–11.1] vs 5.2 [3.6–6.0], p = 0.47) levels did not differ between these groups (Fig 4). Comparably, no differences were seen in markers of inflammation in lung homogenate (Fig S3 in S1 Appendix).

### Pulmonary injury

Plasma compared to standard-volume crystalloid resuscitation did not reduce pulmonary W/D ratios (6.2 [5.3–6.6] vs 6.2 [5.6–7.2], p = 0.54) or cumulative pulmonary histology injury scores (5 [5–7] vs 6 [4–10], p = 0.71) (Fig 5). No differences in any marker of pulmonary injury were seen between any intervention. Distribution of individual organ injury scores are shown in the supplement (Table S2 in S1 Appendix).

## Discussion

This study investigated the effects of plasma resuscitation compared to an equal low volume of crystalloids, as well as to a threefold higher “standard” volume of crystalloid resuscitation, on endothelial injury, glycocalyx degradation, inflammation, NET formation, and organ injury in an experimental rat model of pneumosepsis.

Results suggest a modest protective effect of plasma on markers of endotheliopathy when compared to crystalloids, demonstrated by a trend towards reduced syndecan-1 levels and significantly lower VCAM-1 levels. However, these differences were only observed between the standard-volume crystalloid resuscitation group and the plasma group, with no discernible difference between the low-volume crystalloid and plasma groups. All other markers of endothelial injury and inflammation showed a similar pattern of protection by plasma, albeit not reaching statistical significance. Previously, in a septic shock experimental model, plasma reduced syndecan-1 levels and improved mortality compared to the use of a threefold higher volume of crystalloids [23]. Our current results add to these existing data, suggesting that potential benefits of plasma resuscitation in sepsis seem to be due to a volume-sparing effect rather than to the type of fluid.

In the current model, animals needed volume resuscitation, given that unresuscitated animals do not survive and that the low-volume crystalloid group potentially did not receive an adequate resuscitation volume, as evidenced by decreased MAP and increased mortality in this group. In contrast, plasma infusion had a more favorable effect on hemodynamics, with improved maintenance of MAP compared to both crystalloid groups. Additionally, hemoglobin levels were significantly lower after the resuscitation period in both the standard-volume crystalloid group and the plasma group compared to the low-volume crystalloid group. This likely indicates that plasma infusion resulted in a similar intravascular volume expansion as a threefold higher volume of crystalloids in the current model. Of note, no effects of resuscitation on lactate levels were observed, which may be due to low specificity of lactate as an endpoint of resuscitation [29].

We explored the effects of resuscitation strategies on inflammation and NET formation. There appeared to be a trend towards higher inflammation when a higher-volume of crystalloids was used, albeit not reaching statistical significance. This is in line with previous experimental studies on inflammatory effects of crystalloids [23]. Regarding NET formation, in the current study, we observed no differences in NET formation between any of the intervention groups.

Taking everything together, our data suggest that septic shock patients requiring high resuscitation volumes potentially could benefit from plasma as a volume-sparing fluid, with associated reduced endothelial injury.

No differences in organ injury were seen between any of the groups. This could be attributed to the relatively short duration of the model, which may not have provided sufficient time for multiple organ failure to develop. Lung injury was already severe at the start of the intervention due to inoculation with the bacterium. The severity of the injury may have hampered the possibility of finding differences due to resuscitation strategies. Additionally, there could be a bias due to the observed mortality in the low-volume crystalloid group.

This study has several limitations. The sample size used may not have been sufficient to detect potential differences between interventions due to higher data variability than expected. Regarding the model, a low MAP is a very late symptom and indicates imminent death. Thus, extrapolation to human sepsis is hampered, given that humans are resuscitated to maintain a specific MAP. However, animals had lower blood pressure and hyperlactatemia compared to the healthy controls, representing clinical triggers for resuscitation. Additionally, we did not administer antibiotics or vasopressors in the current model. Therefore, potential positive effects of plasma could be smaller in a model implementing these therapies. Finally, in this model, systemic markers of endothelial and glycocalyx injury were measured as indicators of endothelial damage. Endothelial cells from different organs are known to exhibit heterogeneous responses to pathogenic stimuli [30]. Although our current model primarily was a lung injury model, systemic markers of endothelial injury should be interpreted cautiously as organ-specific endothelial responses may have been masked. However, this was a proof-of-concept study on the effect of plasma versus crystalloids on the endothelium.

## Conclusion

In conclusion, in a pneumosepsis rat model, plasma resuscitation has a modest effect on glycocalyx degradation and endothelial injury, while not improving organ injury when compared to crystalloid resuscitation. However, plasma may allow for volume-sparing resuscitation. Whether septic patients requiring high resuscitation volumes could benefit from plasma as a volume-sparing strategy remains to be determined.

## Supporting information

S1 Appendix Supporting tables and figures. (DOCX)

S2 Appendix Minimal data set. (XLSX)

S3 Appendix ARRIVE guidelines. (PDF)
