# Supplementary material for: Plasma attenuates endothelial injury compared to crystalloids in a ventilated rat pneumosepsis model
Source: PLoS One. 2025 Feb 25;20(2):e0319272. doi: 10.1371/journal.pone.0319272 (PMC11856581; doi:10.1371/journal.pone.0319272)
Supplement: S1 Appendix — (DOCX) [file pone.0319272.s001.docx]

**Plasma attenuates endothelial injury compared to crystalloids in a ventilated rat pneumosepsis model**

Supplemental

Daan P. van den Brink, MD^1, 2^, Derek J.B. Kleinveld, MD, PhD^2, 3^, Chantal A. Polet^2^, Hendrik Veltman, MSc^2^, Joris J.T.H. Roelofs, MD, PhD, Professor^5, 6^, Nina C. Weber, PhD^2, 6^, Nicole .P. Juffermans, MD, PhD, Professor^2, 4^

^1^ Amsterdam UMC, University of Amsterdam, Department of Intensive Care Medicine, Amsterdam, Netherlands

^2^ Amsterdam UMC, University of Amsterdam, Laboratory of Experimental Intensive Care and Anesthesiology, Amsterdam, Netherlands

^3^ Erasmus MC, Erasmus University of Rotterdam, Department of Anesthesiology, Rotterdam, Netherlands

^4^ Erasmus MC, Erasmus University of Rotterdam, Department of Intensive Care Medicine, Rotterdam, Netherlands

^5^ Amsterdam UMC, University of Amsterdam, Department of Pathology, Amsterdam, Netherlands

^6^ Amsterdam UMC, Cardiovascular Sciences, Amsterdam, The Netherlands

**Table of contents:**

| Table S1: Lung injury assessment score list | P3 |
| --- | --- |
| Table S2: Baseline characteristics (T=0) | P4 |
| Figure S1: Kaplan-Meier survival curve | P5 |
| Figure S2: Bacterial outgrowth in lung homogenate | P6 |
| Figure S3: Pulmonary inflammation | P8 |
| Figure S4: Pneumosepsis model animal welfare monitoring Sheet | P8 |

**Table S1: Baseline characteristics (T=0)**

| **Parameter:** | **Healthy**  **No resuscitation**  **(n=6)** | **Pneumosepsis**  **standard-volume crystalloid (n=11)** | **Pneumosepsis**  **Plasma**  **(n=11)** | **Pneumosepsis**  **low-volume crystalloid (n=9)** |
| --- | --- | --- | --- | --- |
| **Weight**  **(g)** | 389  (366 – 415) | 392  (357 – 403) | 391  (380 – 427) | 396  (361 – 404) |
| **Weight loss**  **(%)** | 0.3  (0.9 – -0.1) | 6.6 *  (6.1 – 7.1) | 6.6 *  (6.1 – 8.2) | 6.4 *  (5.8 – 7.6) |
| **Temperature**  **(°C)** | 36.8  (36.4 – 36.8) | 36.6  (35.9 – 36.9) | 36.9  (36.8 – 37.2) | 37.2  (36.9 – 37.3) |
| **MAP**  **(mmHg)** | 129  (123 – 135) | 86 *  (78 – 103) | 86 *  (80 – 96) | 94 *  (90 – 105) |
| **Heart rate**  **(bpm)** | 228  (213 – 230) | 240 *  (232 – 275) | 270 *  (257 – 288) | 262 *  (240 – 276) |
| **Saturation**  **(%)** | 97  (98 – 98) | 93  (92 – 98) | 94  (92 – 98) | 97  (95 – 98) |
| **pH** | 7.40  (7.39 – 7.41) | 7.43 *  (7.42 – 7.46) | 7.46 *  (7.43 – 7.47) | 7.45 *  (7.41 – 7.47) |
| **pCO_2_**  **(mmHg)** | 41.4  (39.3 – 42.6) | 39.4  (37.2 – 45.0) | 38.2  (35.0 – 39.5) | 38.9  (36.0 – 41.4) |
| **HCO_3_^-^**  **(mM)** | 25.0  (23.9 – 25.7) | 26.1  (24.6 – 27.7) | 26.2  (25.6 – 39.5) | 26.5  (24.7 – 27.5) |
| **Base excess**  **(mM)** | -0.1  (-0.6 – 0.7) | 2.2 *  (0.6 – 3.7) | 2.4 *  (1.4 – 3.0) | 2.3 *  (-0.5 – 3.7) |
| **Hb**  **(mM)** | 10.7  (10.5 – 10.8) | 11.5 *  (11.0 – 11.8) | 11.7 *  (11.5- 11.9) | 11.5 *  (11.2 – 11.8) |
| **Glucose**  **(mM)** | 24.3  (23.5 – 25.3) | 13.2 *  (10.9 – 15.0) | 12.0 *  (8.8 – 14.0) | 10.6 *  (8.9 – 12.4) |
| **Lactate**  **(mM)** | 0.82  (0.73 – 0.92) | 1.94 *  (1.64 – 2.30) | 2.01 *  (1.86 – 2.17) | 1.83 *  (1.78 – 2.00) |
| **Leukocytes**  **(*10^9^/L)** | 8.4  (9.8 – 5.6) | 1.7 *  (1.0 – 3.1) | 1.0 *  (0.9 – 1.6) | 2.0 *  (1.3 – 3.3) |
| **Platelets**  **(*10^9^/L)** | 717  (610 – 809) | 569  (391 – 620) | 580  (411 – 790) | 740  (601 – 854) |

*Data are presented as median (interquartile range), *p <0.05 when compared to the healthy control group. Bpm = beats per minute, Hb = hemoglobin, MAP = Mean arterial pressure.*

**Table S2:** **Lung injury assessment scoring list:**

| **Group:** | **Healthy**  **No resuscitation (n=6)** | **Pneumosepsis**  **standard-volume crystalloid (n=11)** | **Pneumosepsis**  **Plasma (n=11)** | **Pneumosepsis**  **low-volume crystalloid (n=9)** |
| --- | --- | --- | --- | --- |
| **Interstitial inflammation** | 1.0  (0.8-1.3) | 2.0*  (2.0-3.0) | 2.0  (1.0-2.0) | 2.0  (0.5-1.5) |
| **Endothelialitis** | 0.0  (0.0-0.0) | 1.0*  (0.0-1.0) | 1.0*  (1.0-2.0) | 1.0*  (0.5-1.5) |
| **Bronchitis** | 0.0  (0.0-0.0) | 2.0*  (1.0-3.0) | 1.0  (0.0-2.0) | 1.0*  (0.0-2.5) |
| **Edema** | 1.0  (0.0-1.3) | 2.0*  (1.0-3.0) | 2.0*  (2.0-3.0) | 2.0  (1.0-2.5) |
| **Thrombi formation** | 0.0  (0.0-0.0) | 0.0  (0.0-0.0) | 0.0  (0.0-0.0) | 0.0  (0.0-0.0) |

*Severity was assessed based on a scale from 0-4 (0, absent; 1, mild; 2, moderate; 3, severe; 4 = very severe). Data are presented as median (interquartile range), *p < 0.05 when compared to the sham group*

**Figure S1: Kaplan-Meier survival curve**

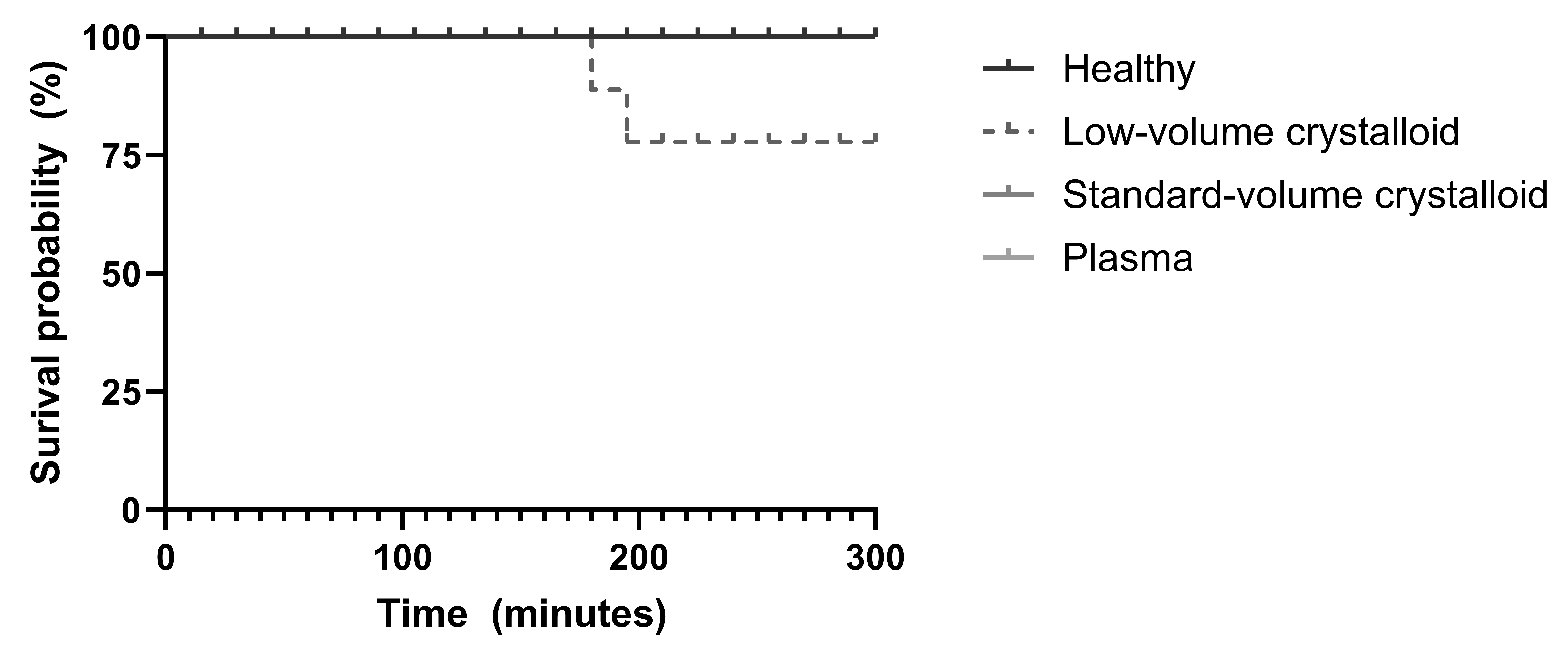


*Kaplan-Meier survival curve and survival table, 2 animals died in the low-volume crystalloid group. No mortality was observed in the other groups.*

**Figure S2:** **Bacterial outgrowth in lung homogenate**


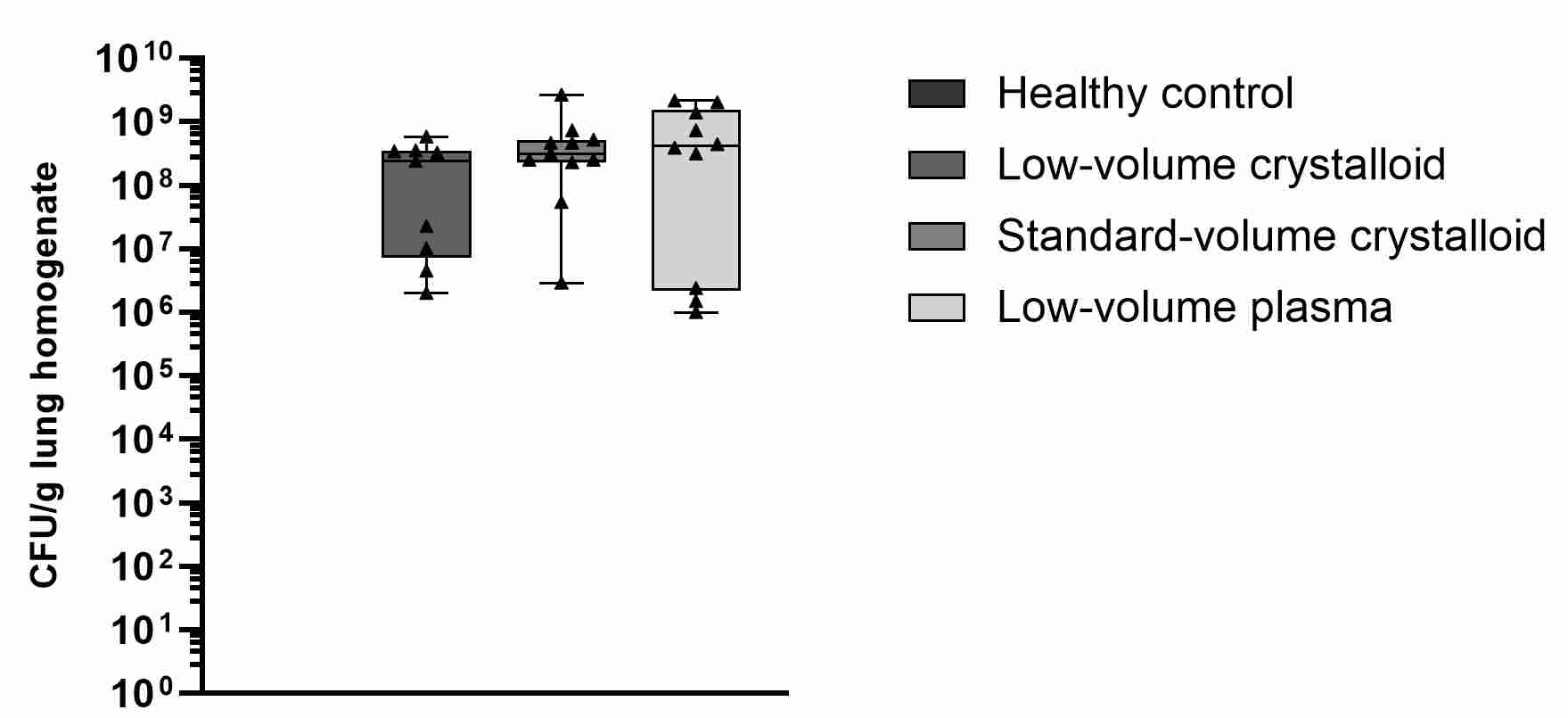


*Data are presented as boxplots with median, interquartile ranges and minimum and maximum values showing all individual data points.*

**Figure S3: Pulmonary inflammation**
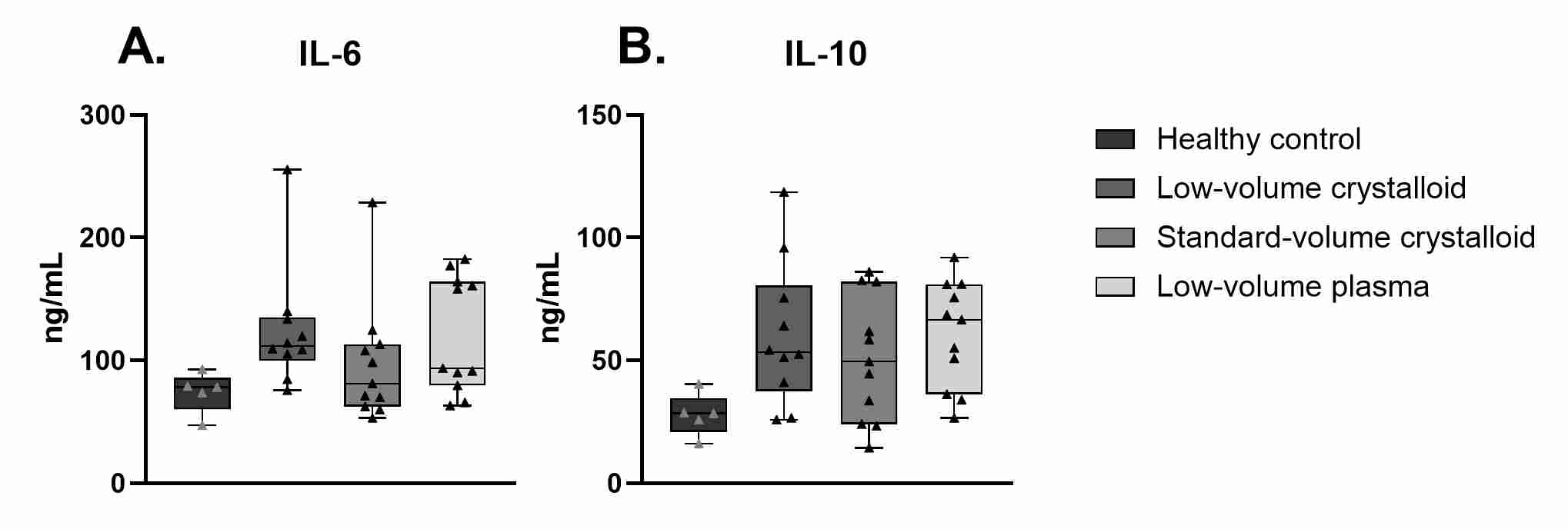


*Data are presented as boxplots with median, interquartile ranges and minimum and maximum values showing all individual data points, IL = Interleukin*

**Figure S4: Pneumosepsis model animal welfare monitoring sheet**

**Animal #:** **Date:**

Fur aspect Actively grooming Dulling of hair coat Rough hair coat Piloerection

Activity Normal Reduced activity disturbed No activity disturbed Nil activity disturbed or
 Reduced activity stimulated stimulated

Behavior Normal, no Slightly hunched, Hunched with stiff Hunched with no
 abd splinting moving freely, mild movement/posture, movement stimulated,
 splinting moderate splinting severe splinting

Face Normal Normal eyelid opening Orbital tightening, Eyelids closed,
 when disturbed moderate grimacing obvious grimacing

Diarrhea None Mild Moderate Severe

Respiratory None Mild dyspnea Moderate dyspnea Severe dyspnea with
distress abdominal breathing

Score 1 2 3 4

A score of 4 in any of the categories results in immediate early termination.

Adapted from:

- Huet O, Ramsey D, Miljavec S, Jenney A, Aubron C, Aprico A, Stefanovic N,Balkau B, Head G, de Haan J, et al.: Ensuring animal welfare while meeting scientific aims using a murine pneumonis model of septic shock. Shock 39(6):488-494, 2013
- Chang R, Holcomb JB, Johansson PI, Pati S, Schreiber MA, Wade CE. Plasma Resuscitation Improved Survival in a Cecal Ligation and Puncture Rat Model of Sepsis. Shock. 2018 Jan;49(1):53-61. doi: 10.1097/SHK.0000000000000918. PMID: 28591008; PMCID: PMC5718978.
